# Intraoperative vascular DIVA surgery reveals angiogenic hotspots in tumor zones of malignant gliomas

**DOI:** 10.1038/srep07958

**Published:** 2015-01-22

**Authors:** Ilker Y. Eyüpoglu, Nirjhar Hore, Zheng Fan, Rolf Buslei, Andreas Merkel, Michael Buchfelder, Nicolai E. Savaskan

**Affiliations:** 1 Department of Neurosurgery, Medical Faculty of the Friedrich Alexander University of Erlangen-Nürnberg (FAU); 2 Department of Neuropathology, Medical Faculty of the Friedrich Alexander University of Erlangen-Nürnberg (FAU)

## Abstract

Malignant gliomas belong to the most threatening tumor entities and are hallmarked by rapid proliferation, hypervascularization and an invasive growth pattern. The primary obstacle in surgical treatment lies in differentiation between healthy and pathological tissue at the tumor margins, where current visualization methods reach their limits. Here, we report on a novel technique (vascular dual intraoperative visualization approach - vDIVA) enabling visualization of different tumor zones (TZ I–III) on the basis of angiogenic hotspots. We investigated glioblastoma patients who underwent 5-ALA fluorescence-guided surgery with simultaneous intraoperative ICG fluorescence angiography. This vDIVA technique revealed hypervascularized areas which were further histologically investigated. Neuropathological assessments revealed tissue areas at the resection margins corresponding to TZ II, and postoperative CD34- and Map2 immunostaining confirmed these angiogenic hotspots to be occupied by glioma cells. Hence, the vascular architecture in this transitional zone could be well differentiated from both primary tumor bulk and healthy brain parenchyma. These data demonstrate that ICG fluorescence angiography improves state-of-the-art glioma surgery techniques and facilitates the future characterization of polyclonal attributes of malignant gliomas.

Gliomas count to the most commonly occurring brain tumors with glioblastomas representing the worst tumor entities in humans^1^. The current treatment strategy includes surgical cytoreduction followed by an adjuvant radio-chemotherapy^2^. Due to the lack of a cure, recurrence is inevitable despite multimodal treatment. As tumor recurrences emerge from remaining tumor cells, it is imperative to maximize the extent of tumor resection to cover at least tumor zones I and II, i.e. the tumor bulk and the tumor infiltration zone^2^,^3^,^4^. Since this is in turn a critical factor in determining survival time in patients with malignant gliomas, the need for identification and intraoperative visualization of these tumor zones through various existing methods cannot be understated^5^,^6^,^7^. The primary obstacle here lies in the precise identification of the extent of tumor infiltration, especially in the border zone^8^. Through intraoperative use of 5-aminolevulinic acid in surgical neuro-oncology, tumor visualization is enabled up to the cellular level^9^. In this biochemical process, tumor cells can be visualized under a blue-light source due to heightened accumulation of protoporphyrin IX particularly in in them. The differentiation ratio between auto-fluorescence and weak fluorescent signal from solitary tumor cells in border zones is especially problematic for the neurosurgeon^10^. One possibility to solve this could lie in visualization approaches of the tumor microenvironment, where neurosurgeons take advantage of the angiogenic properties of malignant gliomas. Through visualization of hypervascularized areas, the so-called angiogenic hotspots, it is possible to identify zones already infiltrated by tumor cells but lacking in critical cell density sufficient to permit clear identification through the fluorescent metabolite. This is possible through the use of indocyanine green (ICG) which is routinely administered intravenously in vascular neurosurgery to control vessel integrity through fluorescence angiography^11^. Furthermore, it is successfully used in tissue and organ perfusion studies^12^,^13^,^14^. In surgical neuro-oncology, this method permits visualization of the tumor microenvironment instead of the tumor itself, i.e. provides indirect evidence of the presence of tumor cells. We conducted here a feasibility study for the novel vascular DIVA (vDIVA) surgery on three confirmed glioblastoma patients. We could demonstrate that vDIVA permits the visualization of individual tumor zones with hypervascularized hotspots through ICG fluorescence angiography.

## Results

### Clinical evidence for the existence of three tumor zones in malignant gliomas

**Figure 1:**
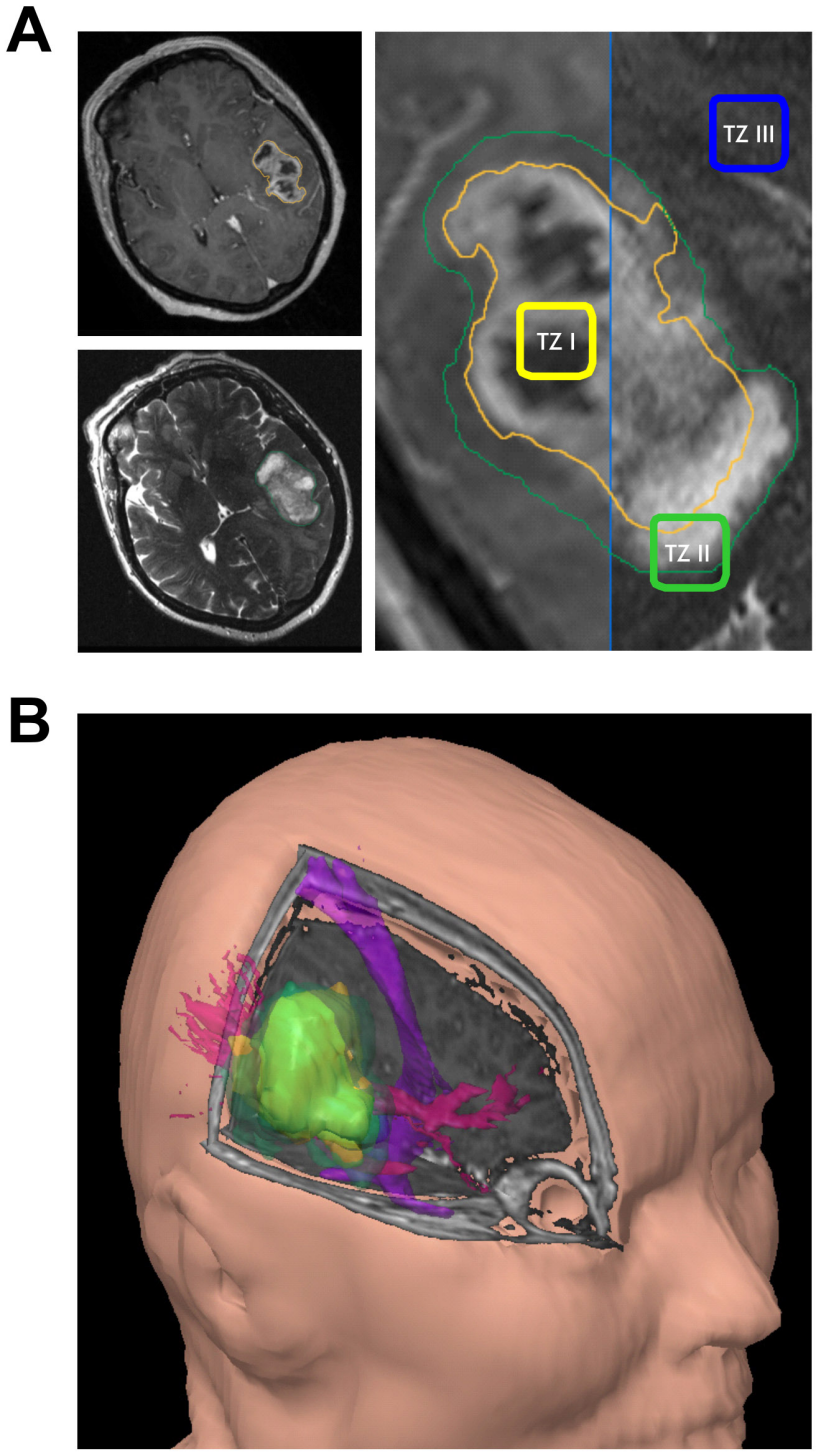
Visualization of differential tumor zones with respect to functionally eloquent areas of the brain. (A), T1 and T2 weighted MR images of a right temporo-dorsal GBM, fused in order to delineate the various tumor zones (TZ): TZ I – corresponding to contrast agent enhancing areas, TZ II – corresponding to the perifocal edema, TZ III – corresponding to apparently healthy brain parenchyma. (B), three dimensional representation of the same tumor in relation to anatomically relevant fiber tracts (pink: optic radiation, purple: pyramidal tract).

One model clarifying the phenomenon of tumor recurrences postulates the existence of three tumor zones^2^. The tumor zone I (TZ I) corresponds to the contrast enhancing areas in the T1-weighted MRI sequences, comprises the main tumor mass and consists of the so-called “core cells” (Fig. 1A). The tumor zone II (TZ II), also known as the peritumoral zone, corresponds to the perifocal edema gets visible in the T2-weighted MRI sequences surrounding the TZ I (Fig. 1A). The tumor cells in this area are called “transitory cells”, since they share many but not all histological und molecular characteristics of the “core cells”. Also seen in this zone are an elevated number of microglia, endothelial cells, and increased angiogenesis where the extent of vascularization is proportional to the degree of malignancy. This region can be assumed to represent the biologically active area of the tumor. The tumor zone III (TZ III) appears to be pathophysiologically inactive as it is characterized by macroscopically almost normal brain parenchyma. However, it is populated by solitary tumor cells – defined as “partisan cells” according to this model – which possess characteristics of tumor stem cells and exhibit pronounced migratory properties. Transposition of the three tumor zones through a three-dimensional reconstruction clearly shows the extent to which the tumor, its microenvironment, and healthy brain parenchyma relate to and interact with each other – and thereby the difficulties in tumor removal in sano (Fig. 1B). Imaging studies permit all three tumor zones to be clearly visualized. The question arising here is whether these three tumor zones can be visualized and differentiated during surgery.

### vDIVA surgery using ICG fluorescence angiography unmasks the TZ II

**Figure 2:**
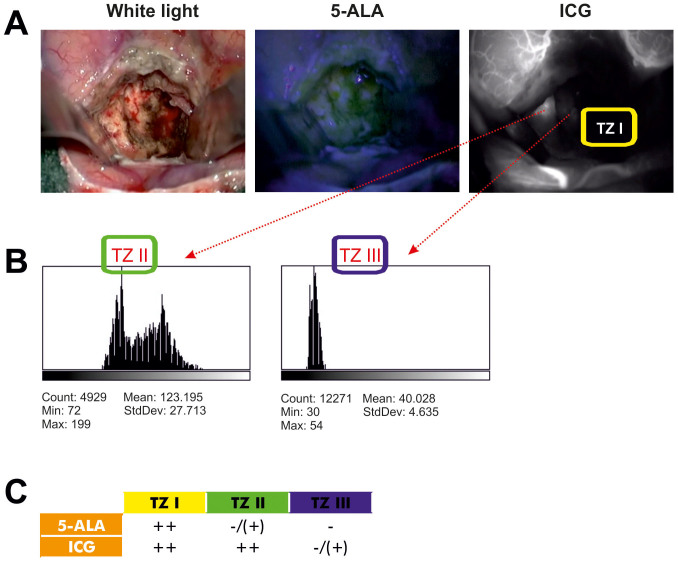
Intraoperative visualization of the tumor zones. (A), The image on the left depicts the tumor cavity following gross total resection in white light. The image in the middle depicts systematic inspection of the same tumor cavity under blue light (5-ALA). The image on the right depicts an area of hypervascularization according to ICG fluorescence angiography in the same tumor cavity where no 5-ALA signal was detectable. (B), Analysis of the intensity of the fluorescence using the open source software ImageJ of the National Institutes of Health. It is clearly seen that sharp increase in fluorescence effectively unmasks the TZ II, whereas TZ III is characterized by drastically reduced fluorescence intensity. (C), We used the following algorithm to differentiate between the three tumor zones: TZ I is 5-ALA and ICG positive, TZ II is 5-ALA negative or weakly positive and ICG positive, TZ III is 5-ALA negative and ICG negative.

**Figure 3:**
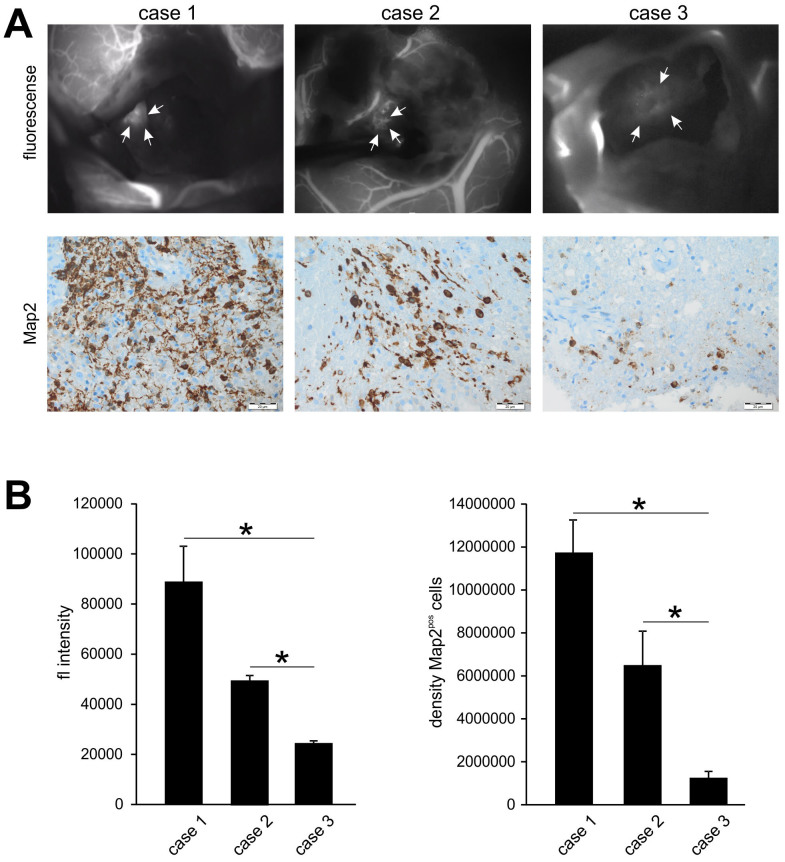
Correlation of ICG positive areas with tumor cell density. (A), The images depict the three cases operated on which the ICG angiography was carried out and the corresponding levels of tumor cell density (Map2) in the positive areas. The extent of Map2 expressing cells showed positive correlation with the intensity of the ICG signal. (B), The graphs depict quantification of the intensity of ICG fluorescence and Map2 signal. Statistical significance was calculated with the Student's t-test (mean ± S.D., *P < 0.05, n = 3 measurements per group).

We carried out 5-ALA fluorescence guided glioblastoma resection until no 5-ALA signal was detectable in the resection cavity – which corresponded to the TZ I – and the brain parenchyma appeared normal under light microscopy. The subsequent ICG fluorescence angiography (Fig. 2A) identified a significantly hypervascularized area at the resection margin (TZ II), which could not be differentiated from surrounding brain parenchyma using light microscopy alone and was negative for 5-ALA signal. The surrounding brain parenchyma (TZ III) appeared unremarkable in all used visualization techniques, i.e. light microscopy, 5-ALA and ICG fluorescence angiography. The intensity of fluorescence in the various regions was quantified with ImageJ (Open Source Software, National Institutes of Health) (Fig. 2B). Through the combination of both approaches, i.e. 5-ALA and ICG, a clear intraoperative distinction of all three different tumor zones is possible (Fig. 2C). Three patients with glioblastoma were operated with the aid of this combined approach consisting of 5-ALA and ICG to test the feasibility of this technique (Fig. 3A, case 1–3). We further investigated the tissue resected from the hypervascularized regions (TZ II) (Fig. 3A). Quantification of both intensity of the ICG signal and density of Map2-expressing tumor cells in these cases (Fig. 3B) showed significant differences in the extents of ICG fluorescence and Map2-staining. The results, nevertheless, consistently documented a positive correlation between ICG fluorescence and Map2-staining: a higher intensity of ICG fluorescence was accompanied by a higher cell density of Map2-expressing cells and vice versa. Therefore, the extent of pathological hypervascularization as determined by the ICG fluorescence clearly corresponds to the tumor load demonstrated by the Map2-staining in a defined area of the brain^15^ — in these three cases the TZ II.

### Histological differentiation between the three tumor zones

**Figure 4:**
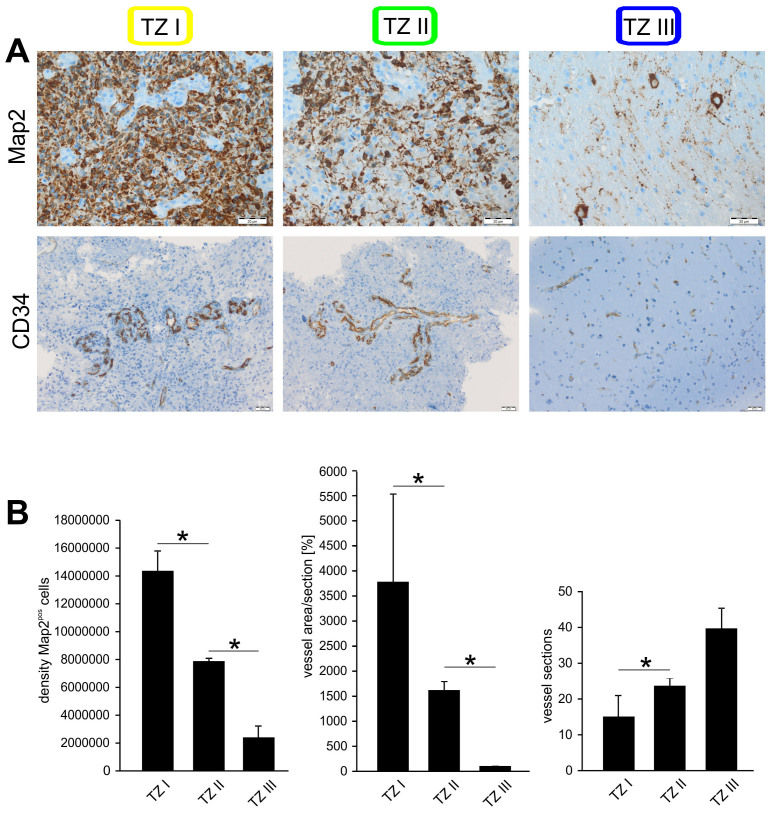
Correlation between Map2 and CD34 expression in the three tumor zones. (A), Patients specimens from the three tumor zones were analyzed with antibodies against Map2 for tumor cell density and CD34, an endothelial marker, for vascular architecture. A marked difference in Map2-staining could be seen between all three tumor zones with decreasing tumor cell density with increasing distance from the center of the main tumor mass, a fact well correlated by investigation of the vascular architecture using CD34 immunohistochemistry. (B), Graphic quantification of the Map2 signal and vascular architecture of the three tumor zones. Although TZ I showed lower vascular density, the vessels appeared significantly thicker than in TZ II and TZ III. Although TZ II showed more vascular sections and narrower vessels than in TZ I, they were more densely packed than in TZ III. Statistical significance was calculated with the Student's t-test (mean ± S.D., *P < 0.05, n = 3 measurements per group).

In a further step, we examined histopathologically the extent to which the three different intraoperatively visualized regions could be differentiated. This was accomplished by immunostaining tissue samples collected out of all three tumor zones from each patient individually using specific antibodies against Map2 and CD34 (Fig. 4A). A significant difference in the number of Map2-positive cells could be seen between all three tumor zones: the farther from the center of the main tumor mass, the fewer the Map2 expressing cell number identified (Fig. 4B). This correlated with the investigation of the vascular architecture using CD34 staining as well. Although TZ I showed no significantly higher vascular density, the vessel walls appeared thickened and plumper indicating higher endothelial proliferation in comparison to TZ II and TZ III (Fig. 4B). Although TZ II showed more vascular sections and narrower vessels than in TZ I, they were more densely packed than in TZ III (Fig. 4B). Therefore, both the CD34-staining and the Map2-staining demonstrated histopathological differences between the three tumor zones.

## Discussion

In glioma surgery, the extent of resection has a significant impact on patient overall survival^2^,^16^,^17^. Since these tumor entities develop endogenously out of brain cells per se^18^,^19^, intraoperative differentiation between healthy and pathological tissue, especially in tumor border zones, poses a serious challenge^8^. One method enabling intraoperative tumor visualization and hence defining the border between healthy and pathological tissue lies in the use of 5-ALA^9^. Tumor cells metabolize this substance, following which they are visible under blue light. Areas occupied by tumor cells in a density less than can be visualized by 5-ALA fluorescence metabolites, therefore, risk remaining in situ when depending on this method alone^10^,^20^,^21^. Through the indirect visualization method evaluated in this feasibility study, the property of angiogenesis of malignant gliomas is exploited and tumor cells which have not reached a minimum density permitting visualization with the naked eye can also be visualized intraoperatively. This means that the property of tumor cells to stimulate angiogenesis is made use of to visualize their microenvironment^22^, thereby unmasking them^2^. Although tumor-occupied areas can be identified through this new method involving ICG as well as through established methods like 5-ALA, their resection can sometimes be accompanied by clinical problems. These areas could be functionally active, so that a resection could reduce tumor mass but cause neurological deficits as well^8^. This means that although ICG and 5-ALA represent powerful tools in the identification of tumor areas, they should be utilized with caution in or close to functionally eloquent areas of the brain – preferably with the assistance of another functional method like intraoperative electrophysiology or functional MR-imaging^23^,^24^.

Although ICG fluorescence angiography has already been utilized in surgery of malignant gliomas, its merits have not been tested to the extent we have. In one such study, pre-resection and post-resection arterial, capillary and venous ICG video-angiographic phases were intraoperatively observed and recorded^6^. Another study analyzed tumoral and peri-tumoral vessels^25^. However, these studies did not investigated on hyperperfusion of peri-tumoral tissue as an aspect of glioma infiltration. The main focus instead was the direct visualization of vessels. We show here for the first time that tumor zones can be directly visualized through vDIVA surgery, which represents an important technical addition to the existing repertoire in surgical neuro-oncology.

Through implementation of the ICG angiography method into DIVA surgery, intraoperative differentiation between various tumor zones and margins is now even more accurate. This acquires importance when it is taken into consideration that increasing evidence points to the fact that glioma cells are not uniform in their exhibition of various properties like proliferation, invasion and capability to induce angiogenesis and immune defense^26^,^27^,^28^. Tumor cells in the core of the tumor mass are more proliferative whereas tumor cells in the transitional zone are more characterized by their ability to induce angiogenesis^27^,^28^. The manner in which tumor cells at a distance from the primary tumor mass behave, i.e. tumor stem cells or partisan cells, and the influence they exert on tumor recurrence is yet to be defined^29^,^30^. This technique in combination with other approaches like 5-ALA guided resection of gliomas now permits intraoperative differentiation between individual tumor areas and therefore between the glioma cell subpopulations. It is clear that intraoperative failure to differentiate between individual tumor areas significantly complicates postoperative molecular biological analyses. In order to evaluate feasibility, we performed a vDIVA surgery on three patients administering the fluorescence dyes ICG and 5-ALA. The ICG-Fluorescence clearly correlated with the immunhistochemical evaluation.

We could show that the intensity of intraoperative fluorescence was directly proportionate to the amount of tumor cells in the immunhistochemical evaluation. In order to reach a conclusion regarding vasculature, a double analysis has to be carried out with visualization through CD34. The size of the vessels was quantified as the surface area and the number of vessel slices evaluated additionally. These data showed that the TZ I is characterized by lower in number but thicker vessels than in the other two zones. Although fewer in number in TZ II i.e. in the invasion zone, the vessels branch much more leading to increased density. TZ III is far easier to identify than the TZ I and II and is characterized by normal vasculature with a much smaller lumen. The data presented here demonstrates immunohistochemical confirmation of the presence of tumor cells in all areas which our novel surgical technique identified as tumor-suspicious zones.

The extent of resection indeed plays a decisive role in boosting the effectiveness of adjuvant treatment and thereby in overall patient survival^17^,^31^,^32^. ICG guided resection permits the resection of tumor areas removed from the main tumor mass, where far fewer cells are detected immunhistochemically. Although supramarginal surgery is presumed to prolong survival time, our new tool should be considered critically in this respect pending further studies to this effect^33^,^34^,^35^,^36^. The sole purpose of this study was to evaluate the feasibility of the ICG method in the resection of malignant gliomas on the basis of the DIVA techique.

At this point we can clearly state that the new vDIVA technique presented here can be easily utilized in glioma surgery, primarily unmasking angiogenic hotspots. Further, these angiogenic hotspots can be regarded as glioma cell niches in the development of recurrences. The extent to which this small amount of additionally resected tumor cells affects overall survival, quality of life or the effectiveness of adjuvant treatment is unclear at present and future investigations will focus on this important matter in randomized follow-up studies.

## Methods

### Patients

A group of three patients with supratentorial GBM underwent elective surgery in the Department of Neurosurgery at the Friedrich-Alexander-University of Erlangen-Nürnberg from February 2012 to March 2012. All three patients were male with a mean age of 59 years (SD: 19.3; range 45–81 years).

### Operation protocol for Angiogenic Hotspots (vDIVA)

MRI sequences utilized were T1-weighted MPRAGE with contrast, T2-weighted, and Diffusion-weighted. Additionally, BOLD functional MRI studies as well as Diffusion Tensor Imaging sequences were integrated to visualize functionally eloquent brain areas according to previously published protocols^8^. All patients received an oral dose of 20 mg/kg bodyweight of a freshly prepared solution of 5-aminolevulinic acid 3 h before induction of anesthesia according to previously published protocols^9^. Solutions were prepared by dissolving 1.5 g of 5-aminolevulinic acid in 50 ml drinking water. A Carl Zeiss OPMI Pentero operating microscope with Xenon white light as well as a blue and green light source for fluorescence imaging was used. Tumor resection was performed using white light alone. At the end of resection, the tumor cavity was systematically inspected using 5-ALA signal to exclude residual tumor. Once the 5-ALA signal was undetectable, administration of ICG was carried out. The ICG module on the microscope was activated and an intravenous bolus of 5 ml of a 5 mg/ml solution of ICG (Pulsion Medical Systems SE, Feldkirchen/Germany) was administered^37^. The additionally resected tissue detected by the ICG was also analyzed by an experienced neuropathologist (R.B.), confirming pathological glioma cell infiltration. Therefore, tissue samples were classified according to the current WHO classification of tumors of the CNS^38^. Immunohistochemical staining was performed as described in detail elsewhere^39^ using a semi-automated benchmark apparatus (Nexes; Ventana, Illkirch, France) and the Ventana DAB staining system following the manufacturer's recommendations. We used a monoclonal antibody against CD34 Class II (1:100; clone: QBEnd-10, DAKO, Glostrup, Denmark); CD34 is expressed in capillary endothelial cells and therefore a useful marker for the identification of blood vessels. All slides were counterstained with haematoxylin. Positive and negative controls were used to validate the staining.

### Statistical methods

Signal intensities as well as cell densities were analyzed using the open source software ImageJ of the National Institutes of Health. Statistical significance was calculated with GraphPad Prism v5.02. A p-value < 0.05 was considered statistically significant in accordance with international conventions. Error bars represent ± S.D. The Student's t-test was used for statistical analysis.

### Ethics

Studies with human tissue were conducted in compliance with the Helsinki Declaration and approved by the Ethics Committee of the Friedrich-Alexander-University of Erlangen-Nürnberg. Written informed consent was obtained from all participants involved in the study. The study complies with the current laws of the Federal Republic of Germany.

### Statements

All methods were carried out in accordance with the approved guidelines. The drawings in the MRI sequences (Fig. 1) were performed by I.Y.E.

## Author Contributions

I.Y.E. conceived and designed the experiments; I.Y.E. performed the described surgical procedures; R.B. performed the neuropathological analyses; N.H., A.M., Z.F., M.B., N.E.S. and I.Y.E. collected and analyzed the data; N.H., Z.F., N.E.S. and I.Y.E. wrote and edited the manuscript. All authors reviewed the manuscript.

## Supplementary Material

Supplementary Information Supplementary Information

Supplementary Information vDIVA Video
