# Supplementary material for: Intraoperative vascular DIVA surgery reveals angiogenic hotspots in tumor zones of malignant gliomas
Source: Sci Rep. 2015 Jan 22;5:7958. doi: 10.1038/srep07958 (PMC4302292; doi:10.1038/srep07958)
Supplement: Supplementary Information [file srep07958-s1.docx]

Intraoperative vascular DIVA surgery reveals

angiogenic hotspots in tumor zones of malignant gliomas

Ilker Y. Eyüpoglu^1,*^, Nirjhar Hore^1^, Zheng Fan^1^, Rolf Buslei^2^,

Andreas Merkel^1^, Michael Buchfelder^1^, Nicolai E. Savaskan^1^

^1^Department of Neurosurgery, ^2^Department of Neuropathology,

Medical Faculty of the Friedrich Alexander University of Erlangen-Nürnberg (FAU)

*Correspondence and requests for materials:

PD Dr. med. Ilker Y. Eyüpoglu

Department of Neurosurgery

Universitätsklinikum Erlangen

University of Erlangen-Nürnberg

Schwabachanlage 6

91054 Erlangen, Germany

Email: ilker.eyupoglu@uk-erlangen.de

or eyupoglu@gmx.net

Tel: +49 9131 85 44756

Fax: +49 9131 85 34569

Supplementary Video legend

*Supplementary Video 1: vDIVA application in an intraoperative setting*

The video shows the essential steps to perform the vDIVA approach by combination of 5-ALA and ICG fluorescence angiography. After craniotomy the tumor was already identified on the brain surface by white light microscopy. Further, the tumor was resected according to the 5-ALA signal (video sequence labeled with 5-ALA). Thereafter, white light and 5-ALA controls were performed indicating complete tumor resection. According to these two standard modalities no further visible tumor cells are identified. However, intraoperative fluorescence angiography (video sequence labeled with ICG) unmasked a hypervascularized zone according to TZ II (video sequence labeled with TZ II), an area which is already occupied by tumor cells and was not visible by 5-ALA and macroscopical aspects. The implementation of ICG into the original DIVA application does not significantly prolong the surgical procedure ^8^.
